# Supplementary material for: Gene Expression Linked to Reepithelialization of Human Skin Wounds
Source: Int J Mol Sci. 2022 Dec 12;23(24):15746. doi: 10.3390/ijms232415746 (PMC9779194; doi:10.3390/ijms232415746)
Supplement: Supplementary file 1 [file ijms-23-15746-s001.zip › Figure S1.pdf]

Figure S1. *IL1B* and *IL6* mRNA Correlation.

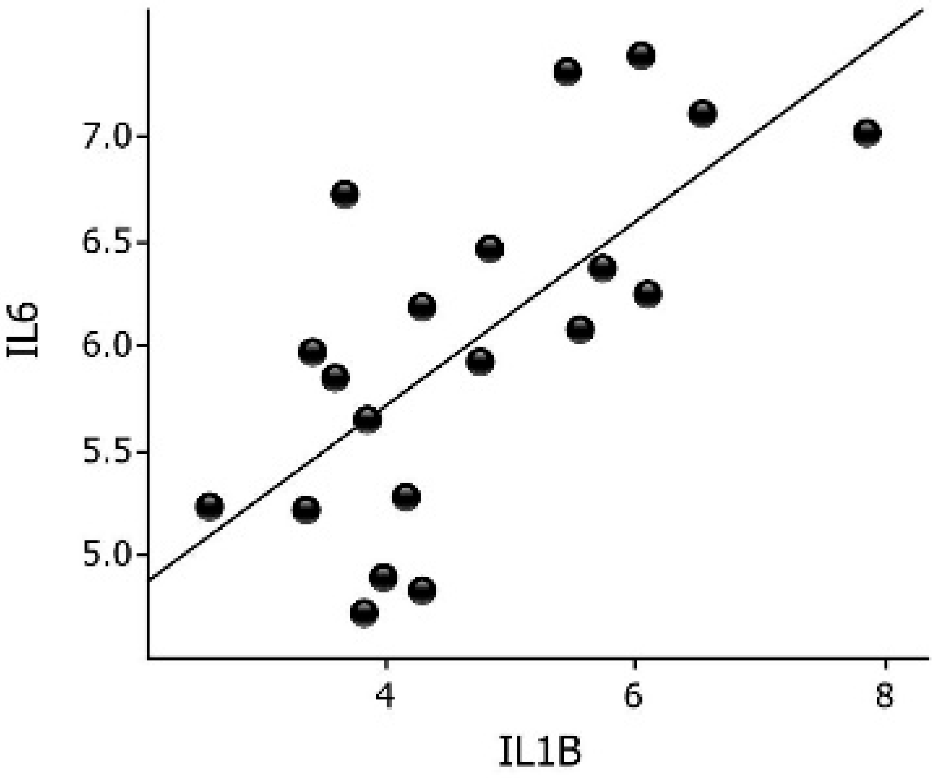

Figure S1. Correlation between *IL1B* and *IL6* mRNA levels in wounds (n = 20). Pearson correlation coefficient: r = 0.69 ($p < 0.001$). Log2-transformed expression values are shown on the y- and x-axes.
