# Supplementary material for: Gene Expression Linked to Reepithelialization of Human Skin Wounds
Source: Int J Mol Sci. 2022 Dec 12;23(24):15746. doi: 10.3390/ijms232415746 (PMC9779194; doi:10.3390/ijms232415746)
Supplement: Supplementary file 1 [file ijms-23-15746-s001.zip › Figure S2.pdf]

Figure S2. Effect of rhIL-6 and rhIL-1β on BrdU incorporation in NHDFs.

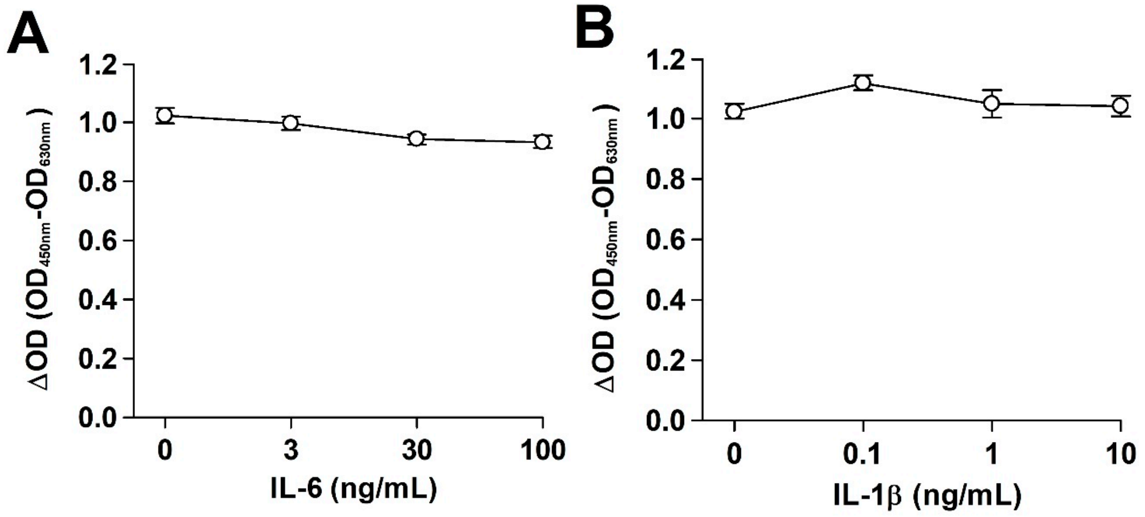

Figure S2. Effect of rhIL-6 (A) and rhIL-1β (B) at the indicated concentration on BrdU incorporation in NHDFs. Cells were seeded into 96-well plates (TPP®, Trasadingen, Switzerland) at 1×10^4 cells per well in complete culture medium and incubated for 72 h. The medium was then replaced with 100 μL DMEM medium with 2.5% FBS and the cells were incubated for another 24 h. IL-6 and IL-1β in 25 μL DMEM with 1 mg/mL BSA were added and cells were incubated for a total of 20 h. After 16 h, 10 μL of BrdU (10 μM, final concentration) was added to each well, except for the background control, to which 10 μL of DMEM alone was added and the cells were incubated for another 4 h. The amount of BrdU incorporation was measured with Roche Cell Proliferation ELISA, BrdU (Cat. No. 11647229001, Sigma-Aldrich, St. Louis, MO, USA). The cells were then washed three times with PBS, fixed, and incubated with a mouse monoclonal anti-BrdU antibody conjugated with peroxidase (1:50 dilution) for 90 min at 37 °C according to the manufacturer's instructions. The immune complex was detected by 100 μL of tetramethyl-benzidine substrate (TMB ONE; Kem-En-Tec Diagnostics, Taastrup, Denmark), and the reaction stopped after 20 min incubation by the addition of 100 μL of 0.2 M H_2SO_4. OD_{450nm} and OD_{630nm} were measured with a microplate reader (800 TS, BioTek Instruments, Winooski, VT, USA). The mean ± SEM of 8 replicates is shown.
