# Supplementary material for: Gene Expression Linked to Reepithelialization of Human Skin Wounds
Source: Int J Mol Sci. 2022 Dec 12;23(24):15746. doi: 10.3390/ijms232415746 (PMC9779194; doi:10.3390/ijms232415746)
Supplement: Supplementary file 1 [file ijms-23-15746-s001.zip › Figure S3.pdf]

Figure S3. Effect of rhIL-6 and rhIL-1β on *MMP3* mRNA levels in NHEKs and NHDFs.

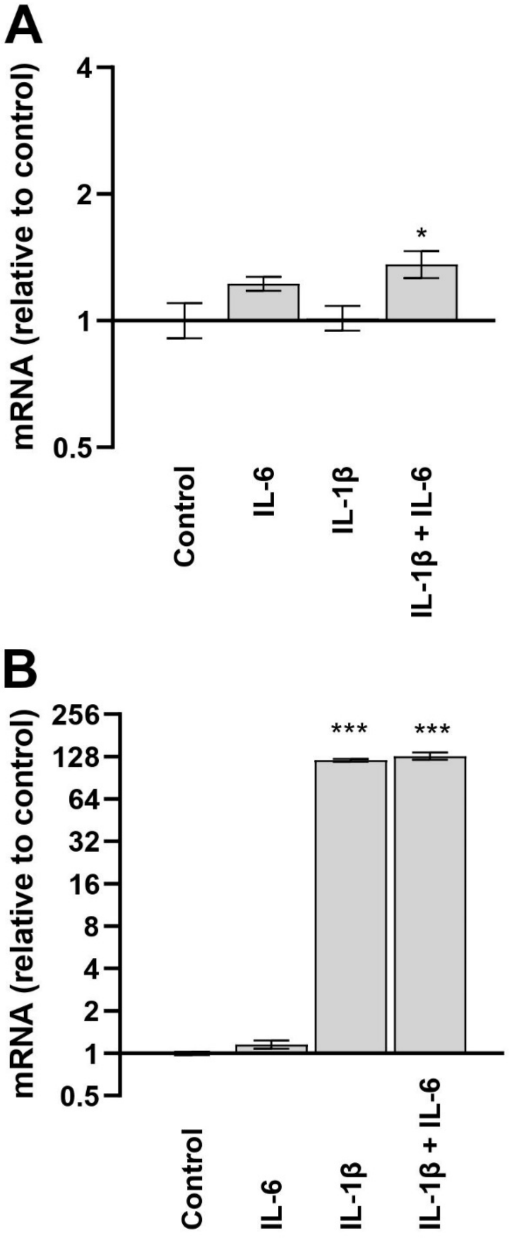

Figure S3. Effect of rhIL-6 (30 ng/mL) and rhIL-1β (1 ng/mL) treatment of NHEKs (A) and NHDFs (B) for 24 h on *MMP3* mRNA. mRNA levels were normalized to *RPLP0*, expressed as fold change relative to control-treated NHEKs and NHDFs. The geometric mean ± back-transformed SEM of 6 replicates is shown. *$p < 0.05$, *** $p < 0.001$ vs. control.
